# A 6-Year Retrospective Study in Surgery Patients with Thyroid Diseases in Mureș County, Romania, Before and During the COVID-19 Pandemic

**DOI:** 10.3390/diagnostics15030287

**Published:** 2025-01-26

**Authors:** Ramona Teodora Cătană, Adela Nechifor-Boila, Ancuța Zăhan, Andreea Deborah Militaru, Georgian-Nicolae Radu, Angela Borda

**Affiliations:** 1 Department of Histology, “George Emil Palade” University of Medicine, Pharmacy, Sciences and Technology of Târgu-Mureş, 38th Gh. Marinescu Street, 540139 Târgu-Mureş, Romania; ramona.catana@umfst.ro (R.T.C.);; 2 Department of Endocrinology, Târgu-Mureş Emergency County Hospital, 50th Gh. Marinescu Street, 540139 Târgu-Mureş, Romania; 3 Department of Pathology, Târgu-Mureş County Hospital, 28th 1 December 1918 Street, 540061 Târgu-Mureş, Romania; george.radu098@gmail.com; 4 Department of Pathology, Târgu-Mureş Emergency County Hospital, 50th Gh. Marinescu Street, 540139 Târgu-Mureş, Romania

**Keywords:** COVID-19, pandemic, thyroid, cancer, impact

## Abstract

**Background/Objectives:** In this study, we aimed to assess the impact of the COVID-19 (Coronavirus Disease 2019) pandemic period on the time trend prevalence of thyroid pathology in a university hospital in Romania. **Methods:** We performed a 6-year retrospective study (2017–2022) including all patients who underwent thyroid surgery, registered in the Pathology Department, Emergency County Hospital, Târgu-Mureș, Romania (n = 971). Thyroid lesions were grouped into three major categories: (1) benign, non-tumoral; (2) benign, tumoral; and (3) malignant, tumoral. To assess the impact of the COVID-19 pandemic on the annual rate of thyroid surgeries and thyroid pathology, data were analyzed in comparison: before COVID-19 (2017–2019) versus the COVID-19 (2020–2022) period. **Results:** A significant decrease in the mean number of thyroid specimens per year was observed in the COVID-19 period compared to the previous period (131 versus 192 cases, *p* = 0.0023). Thyroid benign lesions were the most frequent, but their prevalence was significantly lower during the pandemic period (50.8%) compared to the previous period (58.6%) (*p* = 0.017). Benign tumors were rare, revealing similar occurrence rates in both periods. By contrast, the annual rate of malignant tumors increased significantly during the COVID-19 period (26.3% versus 35.4%, *p* = 0.002), the most common histopathological type being papillary thyroid carcinoma. **Conclusions:** Along with the COVID-19 pandemic (starting in the year 2020), due to reduced access to medical investigations, many thyroid cancers remained unoperated in our hospital. Consequently, this has led to an increased prevalence of malignant cases in the years that came after.

## 1. Introduction

The COVID-19 (Coronavirus Disease 2019) pandemic, declared by the WHO (World Health Organization) on 11 March 2020, produced major changes worldwide in many different spheres of society, including the economy and public health [1,2]. The novel SARS-CoV-2 virus was first identified in an outbreak in the Chinese city of Wuhan in December 2019 [3]. COVID-19, also known as coronavirus disease, is the infectious illness caused by the SARS-CoV-2 virus, characterized by dry cough, loss of taste or smell, lung complications such as pneumonia, and, in the most severe cases, acute respiratory distress syndrome. The virus has spread rapidly to more than 215 countries. By March 2024, it had infected more than 704 million people and caused the death of more than 7 million [4]. The new pandemic situation that allocated the medical resources to treat patients with severe forms of SARS-CoV-2 infection imposed measures with a significant impact on public access to health services and on the quality of curative and preventive care provided for other conditions [5].

Literature data focused on the impact of the COVID-19 pandemic on thyroid pathology with a particular interest in neoplastic disorders are scarce [6,7,8]. SARS-CoV-2 infection has been associated with thyroid disorders that can manifest biochemically as thyrotoxicosis, hypothyroidism, as well as nonthyroidal illness syndrome. The virus can affect thyroid function either through a primary thyroid injury or through a secondary pituitary or hypothalamic lesion. The virus enters host cells using a molecular complex involving angiotensin-converting enzyme 2 (ACE2) and transmembrane protease serine 2 (TMPRSS2), both more extensively expressed in the thyroid gland than in the lungs [9,10,11,12]. The mechanisms involved in thyroid dysfunctions could be caused either by the direct effect of the virus or by an indirect effect through a systemic abnormality of the inflammatory immune response caused by SARS-CoV-2 infection [10,12].

Pathologies affecting the thyroid gland and their management can significantly influence patients’ long-term well-being. Of these, the most threatening is thyroid cancer, which is the most common endocrine malignancy, accounting for ~2.1% of all cancer diagnoses worldwide.

The incidence of thyroid cancer has significantly increased over the past few decades. Most of this increase is attributed to papillary thyroid cancer (PTC). This trend has been observed in higher-income countries (Republic of Korea, Canada, Italy, France, Israel, Croatia, Austria, U.S.A.), middle-income countries, and even island nations and territories (Cyprus, Cabo Verde, French Polynesia) [13]. In the United States, thyroid cancer is estimated to be the 13th most commonly diagnosed cancer [13], and in urban areas in China, it ranks 4th among all malignant tumors in women [14]. Currently, one in 55 U.S.A. women and one in 149 U.S.A. men are expected to be diagnosed with thyroid cancer during their lifetime [13]. The occurrence of thyroid cancer has been linked to radiation exposure, iodine intake, diabetes, obesity, Hashimoto thyroiditis, exogenous estrogen use, and dietary choices [15]. Moreover, the general population’s easy access to medical imaging investigation methods has led to the discovery of small, indolent thyroid microcarcinomas.

In this study, we aimed to assess the impact of the COVID-19 pandemic period on the time trend prevalence of thyroid pathology in a university hospital in Romania, with special emphasis on the diagnosis and outcome of thyroid cancer.

## 2. Materials and Methods

### 2.1. Database and Study Design

We performed a 6-year retrospective study including all patients who underwent thyroid surgery and who were registered in the Pathology Department, Emergency County Hospital, Târgu-Mureș, Romania, between January 2017 and December 2022, a time period centered by the year 2020, when the COVID-19 pandemic broke out. In 1999, Romania was considered an iodine-deficient country, but starting in 2002, National Guidelines regarding universal iodization of salt intended for human consumption, animal feed, and use in the food industry were implemented. Fifteen years after this government decision, Romania may no longer be considered an iodine-deficient region.

The Ethics Committee of the Emergency County Hospital, Târgu-Mureş approved the study (Letter of Approval no. 1727/26 January 2023).

### 2.2. Pathological Data and Study Groups

All patients who underwent thyroid surgery (total thyroidectomy or lobectomy) between January 2017 and December 2022 were included in the study. Patient demographics (age, gender) and pathological data (complete histopathological diagnosis, tumor histology, lymph node involvement, extra-thyroidal extension, etc.) were all retrieved from institutional database registries and pathological reports.

To assess the impact of the COVID-19 pandemic on the annual rate of thyroid surgeries and thyroid pathology in our department, data were analyzed in comparison: before COVID-19 (3 years, January 2017–December 2019) versus the COVID-19 (3 years, January 2020–December 2022) period. Different types of thyroid lesions were divided into 3 separate groups: 1. benign non-tumoral pathology (encompassing nodular and diffuse goiter, Basedow–Graves disease, autoimmune thyroiditis, subacute thyroiditis, thyroid tuberculosis, abscess and cysts), 2. benign tumors, 3. malignant tumors.

Thyroid sampling was performed according to international guidelines [16], with at least 3 sections taken for each lobe in the case of benign non-tumoral lesions and following specific recommendations in the case of tumoral lesions. The histological type of tumor was established according to the 2017 WHO Classification of Tumors of Endocrine Organs [17]. Benign tumors encompassed follicular adenoma (FA) cases and variants. Other entities included hyalinizing trabecular tumors and encapsulated follicular-patterned thyroid tumors; thyroid tumors of uncertain malignant potential (TT-UMP) and noninvasive follicular thyroid neoplasms with papillary-like nuclear features (NIFTP) were also included in this category due to their indolent, benign biologic behavior, as well as post-surgery management that is similar to FAs, which is clinical follow-up only. Malignant tumors included: (1) PTC cases (conventional and variants: follicular; encapsulated; papillary microcarcinoma; oncocytic Warthin-like; tall-cell; hobnail; solid/trabecular; and diffuse sclerosing), (2) follicular thyroid carcinoma (FTC) cases, (3) Hürthle cell carcinoma cases, (4) poorly differentiated thyroid carcinoma cases, (5) anaplastic thyroid carcinoma cases, (6) medullary thyroid carcinoma cases, (7) metastasis, and (8) rare thyroid malignant tumor cases (limphoma, angiosarcoma, plasmacytoma). PTCs with poorly differentiated areas were considered as poorly differentiated thyroid carcinomas since the presence of poorly differentiated areas in a background of well-differentiated thyroid carcinoma carries a worse prognosis.

### 2.3. Statistical Analysis

Statistical analysis was performed using Statistical Package for Social Sciences (SPSS, version 22, Chicago, IL, USA), Excel 2021, and GraphPad Prism 7 programs. Descriptive statistical analyses were performed. The data were expressed as nominal or quantitative variables. The nominal variables were characterized by means of frequencies. The frequencies of the nominal variables were compared using the Chi-squared test. The normal distribution of continuous variables was assessed using the Kolmogorov–Smirnov test or histograms. The distributed continuous variables were expressed as mean +/− standard deviation (SD). The Student test was applied to compare continuous values with Gaussian distribution. The ANOVA test was used to compare several mean values. We analyzed the data in a comparative way between two periods: before COVID-19 (3 years, 2017–2019) and during the COVID-19 period (3 years, 2020–2022). To compare parametric data, the exact Fisher test was applied. The level of statistical significance was set at *p* < 0.05.

## 3. Results

### 3.1. Patient Characteristics

A total of 971 thyroid specimens were registered in our department over the study period: 578 (59.5%) cases before the COVID-19 period and 393 (40.5%) cases in the COVID-19 period. Among the 578 cases registered between 2017 and 2019, 510 (88.1%) were women and 68 (11.9%) were men, the women/men (W/M) ratio being 7.5/1; the mean age at diagnosis was 52.37 years-old (range 18–85 years). With regard to the second study period (during COVID-19), among the 393 patients submitted to surgery, 339 (86.3%) were women and 54 (13.7%) were men (W/M ratio: 6.2/1); the mean age at diagnosis was 52.05 years old (range 7–86 years). Interesting to note was that men who underwent surgery during the COVID-19 pandemic were significantly older compared to those who underwent surgery before the COVID-19 pandemic (60 years old versus 54 years old, *p* = 0.002).

**Figure 1:**
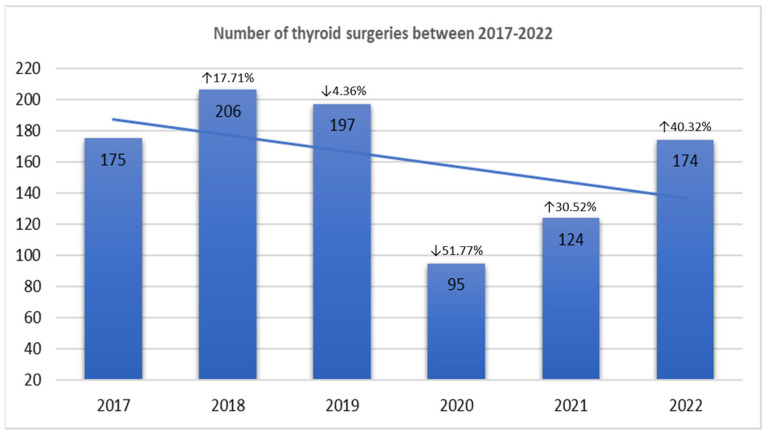
Number of thyroid surgeries per year and their time trend over the study period (between 2017 and 2022); the percentages reflect either the increase or the reduction with regard to the previous year. The most spectacular reduction in the number of thyroid surgeries occurred in 2020 (n = 95), corresponding to a reduction of 51.77% in thyroid surgeries performed in 2019.

A significant decrease in the mean number of thyroid specimens per year was observed in the COVID-19 period compared to the previous period (131 cases [95% CI: 31.73–230.3] versus 192 cases [95% CI: 153.1–232.3], *p* = 0.0023). The most significant reduction in the number of thyroid surgeries occurred in 2020 (n = 95 versus n = 197 cases in the preceding year, 2019, *p* = 0.001) (Figure 1). In 2022, the number of thyroid surgeries was again similar to those performed in 2017, prior to the COVID-19 pandemic.

Table 1 summarizes the frequency of different thyroid lesions/tumors in comparison before COVID-19 (2017–2019) versus the COVID-19 (2020–2022) period. During both study periods, the highest prevalence was observed for benign non-tumoral thyroid lesions, followed by malignant and benign thyroid tumors.

**Figure 2:**
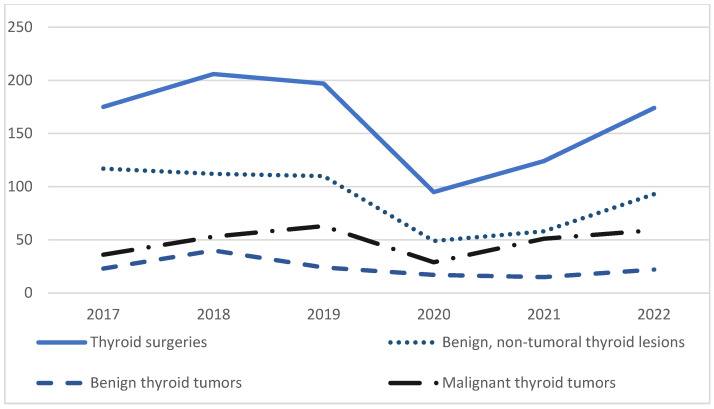
Time trend prevalence of the three study groups (benign non-tumoral lesions, benign tumors, and malignant tumors, respectively) over the study period (between 2017 and 2022), with respect to the number of thyroid surgeries.

Figure 2 illustrates the time trend prevalence of the three study groups (benign non-tumoral lesions, benign and malignant tumors, respectively) over the entire study period (between 2017 and 2022), with respect to the number of thyroid surgeries.

#### 3.1.1. Benign Non-Tumoral Pathology Group

Benign non-tumoral thyroid lesions represented more than half of the cases in our study population. However, a consistent reduction in their percentage (among all thyroid lesion types) was observed during the pandemic period (50.8%) compared to the previous period (58.6%) (*p* = 0.017).

The demographic characteristics of the patients belonging to this group are listed in Table 2. In both study periods, most of the cases occurred in women, but a slight decreasing trend of the gender gap was observed between 2020 and 2022 (W/M ratio 9.2/1 versus 7.3/1). The patients’ average age was 53 years old for both periods. Interestingly, men who underwent surgery during the pandemic were significantly older compared to those admitted for surgical treatment between 2017 and 2019 (60 years old versus 54 years old, *p* = 0.002).

#### 3.1.2. Benign Thyroid Tumors Group

Benign thyroid tumors were rare, revealing similar occurrence rates in both periods: 87 cases (15.1%) in the first period versus 54 cases (13.8%) in the second period (*p* = 0.579).

The demographic characteristics of the patients belonging to this group are listed in Table 2. No significant differences were observed between the two study periods. The patients’ mean age was 49 and 51 years old between 2017 and 2019 and 2020 and 2022, respectively. In both study periods, most of the cases occurred in women (W/M ratio 7.7/1 versus 6.8/1).

Table 1 summarizes the frequencies of benign thyroid tumors and their different histologies in comparison before COVID-19 (2017–2019) versus during the COVID-19 (2020–2022) period. The most frequent tumor type in this category was FA [52.9% (2017–2019) versus 40.7% (2020–2022), *p* = 0.170], followed by encapsulated follicular-patterned thyroid tumors (non-invasive) (32.2% versus 44.4%, *p* = 0.154). Even though variations in the proportion of these cases were observed between the two study periods (decreasing trend for adenomas and increasing trend for encapsulated follicular-patterned thyroid tumors), no statistically significant differences in the annual rates were recorded.

Hürthle cell adenoma cases were less frequent and revealed a similar prevalence trend in the two study periods (14.9% versus 12.9%, *p* = 0.808); hyalinizing trabecular tumor cases were extremely rare.

#### 3.1.3. Malignant Thyroid Tumors Group

Two hundred ninety-one (n = 291) cases of malignant thyroid tumors were registered in our department over the study period. The annual rate of malignant tumors increased significantly during the COVID-19 period (35.4%, CI (95%) [11.91 to 40.27] versus 26.3%, *p* = 0.002), the most common histopathological type being PTC. The highest prevalence was observed in 2021 (41.1%) and 2022 (33.9%). Three years after the pandemic outbreak, the cancer prevalence remains higher than the average rate observed in the pre-COVID-19 years (Figure 2).

Regarding demographic characteristics of the study cases (Table 2), our data revealed that male patients with malignant thyroid tumors during the COVID-19 period were younger compared to men diagnosed in the pre-COVID-19 period (48.87 years old versus 54.65 years old), although the difference did not reach statistical difference (*p* = 0.186). We also noticed that during the COVID-19 period, men diagnosed with malignant thyroid tumors were significantly younger than men diagnosed with benign, non-tumoral lesions in the same period (48.47 versus 60.83 years old, *p* = 0.002). The female gender was predominant, but compared to the benign non-tumoral and tumoral lesions, the gender disparity was lower [the W/M ratio was 4.6/1 versus 9.2/1 or 7.7/1 (2017–2019) and 5.3/1 versus 7.3/1 or 6.8/1 (2020–2023), respectively.

The prevalence of different types of malignant thyroid tumors is shown in Table 1 in comparison before COVID-19 versus during the COVID-19 period. The most common type of thyroid cancer was PTC in both study periods, accounting for 118 (77.6%) and 124 (89.2%) cases, respectively. A statistically significant increase in the annual rate of PTC was observed in the COVID-19 period compared to the previous years (*p* = 0.011). Other malignant tumor types (FTC, Hurthle cell carcinoma, poorly differentiated carcinoma, anaplastic carcinoma) were rare in both periods. A significant decrease in the prevalence of medullary thyroid carcinoma, a rare tumor in general, was observed during the second period compared to the 2017–2019 interval (4 cases—2.9% versus 15 cases—9.9%, *p* = 0.017).

When analyzing the histology of PTC and its variants (Table 1), for both study periods, most PTC cases corresponded to conventional PTCs (51.8% versus 41.2%). The proportion of small thyroid tumors, namely papillary thyroid microcarcinomas (≤1 cm), remained constant throughout the two studied periods (19.5% versus 20.9%, *p* = 0.873).

## 4. Discussion

Since the outbreak of the COVID-19 pandemic, many scientific articles have been published about the SARS-CoV-2 virus and its related pathology [2,18,19]. Nevertheless, there are still few that have assessed the impact of this pandemic situation on the management of other pathologies that do not represent life-threatening emergencies (e.g., thyroid diseases). The aim of the present study was to investigate the impact of the COVID-19 pandemic period on the time trend prevalence of thyroid pathology in a university hospital in Romania (Mureș County Emergency Hospital), with special emphasis on the diagnosis and outcome of thyroid cancer.

It is well known that the global prevalence of thyroid disease is high and continues to rise, with thyroid goiter being by far the most common form of thyroid disorder. Most thyroid pathologies do not require surgical intervention and can be effectively managed through alternative approaches (oral treatment, clinical or imaging follow-up), and only a limited ratio of patients diagnosed with thyroid diseases experience oncological or clinically severe thyroid disorders. Surgical considerations are recommended in cases associated with symptoms of cervical compression, hyperthyroidism that is refractory to treatment, and high suspicion of malignancy.

During the pandemic crisis, social distancing and/or social isolation were the most recommended and even imposed protection measures against the spread of the Sars-CoV-2 virus [20]. The access of the general population to medical services was limited [21,22,23,24]. According to WHO guidelines, the medical services were classified as essential or non-essential, which allowed resources to be redirected to the pandemic response. This has caused cancellations or delays in elective and non-urgent procedures, like most of those involving thyroid pathology. Other important reasons why patients avoided medical services were: the fear of contagion [21,25,26], the perception that the medical services would not be of a high standard [21,27], and, not in the end, the worsening socioeconomic situation of the population. Moreover, there was a rapid conversion of the classic face-to-face consultation to telemedicine (online consultation, telephone, video call, etc.), which accounted for up to 80% of all consultations, according to published data [28]. Remote examination of the patient with thyroid nodules, without a physical and ultrasound examination of the thyroid, is defective and involves the risk of missing the diagnosis of nodules that require surgical treatment. Further, the pandemic has also led to a drastic reduction in the frequency of fine needle aspiration biopsies of the thyroid nodules. During the COVID-19 pandemic, this procedure has become extremely rare, performed only for highly selected cases, which has led to a decrease of up to 98% in its frequency, as reported by previous studies [29].

In line with all these arguments, the results of our study also revealed a significant decrease in the number of thyroid surgeries performed during the COVID-19 period compared to the previous period. This decrease was particularly notable in the year 2020, the hotspot of the COVID-19 pandemic. Nevertheless, the annual rate of malignant tumors in our study increased significantly during the COVID-19 period (*p* = 0.002), with PTC being the most common histopathological type. The highest prevalence of thyroid cancer was observed in 2021 (41.1%), the first year following the pandemic outbreak.

Our results are similar to those recently reported by other Romanian authors analyzing data from different territorial regions in Romania [30,31]. In their study, Popa O et al. also observed a significantly higher prevalence of more aggressive pathological thyroid tumor types in the POSTCOVID group (17.7%) compared to the PRECOVID group (7.6%; *p* = 0.0006). In their study performed in South Korea (2019–2021), Seong Hoon Kim et al. reported a persistent increasing trend in the incidence of thyroid malignant tumors despite the reduced thyroid surgical interventions [32]. Additionally, the authors of this study also emphasized that the proportion of worse prognostic factors (extrathyroidal extension, lymphatic invasion, vascular invasion, and neck lymph node metastasis) was significantly higher among PTC patients diagnosed during the COVID-19 outbreak. A plausible explanation could be the delay in diagnosis and surgical interventions as healthcare systems were reorganized in accordance with the pandemic-related challenges, and patients may have faced delays in seeking medical attention, diagnostic procedures, and elective surgeries.

The proportion of small thyroid tumors, namely papillary thyroid microcarcinomas (≤1 cm), remained constant throughout the two studied periods, which can be explained by the indolent clinical behavior characteristic of these tumors. Thyroid microcarcinomas generally do not cause symptoms or complications that require immediate or urgent surgery. In many cases, these tumors are discovered accidentally, usually during imaging investigations for other conditions or during routine exams. Indeed, studies have reported an increasing thyroid tumor size in cases diagnosed in the pandemic study groups (4.0 ± 1.9 cm—2020 year, 4.3 ± 2.3 cm—2021 year) compared to the group before COVID-19 (3.5 ± 2.2 cm—2019 year) [32]. The more frequent diagnosis of larger tumors during the COVID-19 period may be associated with the onset of cervical compressive symptoms, leading to prompt surgical intervention without significant delays. On the other hand, cervical discomfort experienced by some individuals during the COVID-19 pandemic might have been associated with SARS-CoV-2 infection. This connection could have led to the prompt diagnosis of thyroid tumors, subsequently resulting in timely referrals to surgical services.

It has been reported that the thyroid gland is vulnerable to SARS-CoV-2 infection [10,12,33]. Many studies have analyzed the functional aspect of the thyroid and have observed thyroid dysfunctions in patients diagnosed with SARS-CoV-2 infections [6,7,10,34,35]. Histopathological studies on thyroid specimens from SARS-CoV-2-infected patients have revealed severe destruction of parafollicular and follicular epithelial cells, leading to follicle rupture [9]. However, no study has demonstrated a direct association between SARS-CoV-2 infection and thyroid cancer. The increased proportion of malignant thyroid tumors during the COVID-19 period could be related to improved screening of patients with thyroid nodules who were referred for surgical evaluation in that period. Moreover, the patients seeking medical services during the COVID-19 period had specific and urgent reasons rather than pursuing routine visits. The surge in cases of malignant tumors during the pandemic years, when nearly all medical services were limited, does not represent a real increase. Rather, it reflects the fact that a considerable number of tumor cases were deferred and diagnosed later due to the reorganization of the health system and the prioritization of the COVID-19 crisis instead of chronic diseases.

With regard to the gender distribution of thyroid pathology, we did not observe significant changes before and after the appearance of the SARS-CoV-2 virus. However, although women were the most affected in both periods, the gender disparity (W/M ratio) was lower in the malignant thyroid tumors group [4.6/1 vs. 7.7/1 vs. 9.2/1 (2017–2019) and 5.3/1 vs. 6.8/1 vs. 7.3/1 (2020–2023), respectively]. Thyroid cancer has always been more common in women than men. A large study (1983–2017, USA) aiming to examine the gender differences in thyroid cancer incidence has reported a W/M ratio of 4.28/1 in patients diagnosed with small localized PTCs, which was reduced to 2.41/1 when larger and higher stage PTCs were considered [36]. In a meta-analysis of 12 pooled study populations, the autopsy prevalence of subclinical PTC was 14.0% in women and 10.8% in men [36]. Some experts have proposed a link between female hormonal factors and thyroid cancer [37,38]. Systematic review, in vitro, and experimental studies using animal models aiming to assess a possible link between estrogens and thyroid cancer have demonstrated weak and discordant data and no consistent association between thyroid cancer risk and estrogens [37,39,40]. Since the incidence rates are similar for both women and men, as cancer lethality increases, it has been suggested that higher rates of thyroid cancer could be attributed to women’s greater engagement in healthcare and to gender differences in healthcare utilization and patterns of clinical thinking in which women are subject to over detection and men may be at risk of under detection. In line with these observations, in our study, we noticed a decreasing trend in the W/M ratio as the histopathological diagnosis became more and more severe. The highest W/M ratio was found in the benign non-tumoral lesions group, while the lowest W/M ratio was seen in the malignant tumors group, in both time periods.

In our study population, we also found that during the COVID-19 period, male patients diagnosed with malignant thyroid tumors were younger compared to men diagnosed with malignant tumors in the pre-COVID-19 period. This observation might be attributed to the fact that surgeons may have selected younger patients to minimize the risk of serious illness from COVID-19 or that the older patients opted against surgery hospitalization in a period when hospitals were considered hotspots for infection.

Thyroid cancer is the only type of cancer in which a patient’s age is used as a prognostic factor included in the TNM staging system. With the same degree of cancer involvement, patients aged <55 have a distinctly different prognosis than those aged >55 [41]. The reasons for this age-based different prognosis are not entirely clear, but it is assumed that older age, in addition to the changes in the thyroid tissue, is also involved in the loss of radioiodine avidity [41].

## 5. Conclusions

Our study highlights how unexpected crises, like the COVID-19 pandemic, can have far-reaching effects on healthcare practices beyond their direct implications, herein including the management and prevalence trend of thyroid pathology.

After the onset of the COVID-19 pandemic, at the beginning of 2020, the number of thyroid surgeries decreased dramatically in our hospital and were mainly reserved for elective cases with high suspicion of malignancy. Further, due to reduced access to medical investigations by the end of 2020, many thyroid cancers remained unoperated. Consequently, this has led to an increased prevalence of malignant cases in the years that came after. This aspect should be monitored carefully because the delay in screening programs and planned examinations impacts the outcome of thyroid cancer patients. Further, larger studies are needed to confirm our observations.

## Figures and Tables

**Table 1:** Distribution of different types of thyroid lesions and tumors in comparison, before COVID-19 (2017–2019) versus the COVID-19 (2020–2022) period.

	2017–2019 n (%)	2020–2022 n (%)	*p* *
Benign, non-tumoral thyroid lesions	339 (56.8)	200 (50.8)	0.017
Nodular goiter	268 (79)	169 (84.5)	0.139
Diffuse goiter	13 (3.8)	2 (1)	0.060
Basedow–Graves disease	24 (7.1)	11 (5.5)	0.588
Autoimmune thyroiditis	27 (7.9)	15 (7.5)	0.990
Cysts	5 (1.5)	2 (1)	0.990
Subacute thyroiditis	1 (0.3)	0	-
Thyroid tuberculosis	1 (0.3)	0	-
Thyroid abscess	0	1 (0.5)	-
Benign thyroid tumors	87 (15.1)	54 (13.7)	0.579
Follicular adenoma	46 (52.9)	22 (40.7)	0.170
Hürthle cell adenoma	13 (14.9)	7 (12.9)	0.808
Hyalinizing trabecular tumor	0	1	-
Other encapsulated follicular-patterned thyroid tumors (non-invasive)	28 (32.2)	24 (44.4)	0.154
TT-UMP	9 (32.2)	10 (41.6)	0.151
NIFTP	19 (67.8)	14 (58.4)	0.782
Malignant thyroid tumors	152 (26.3)	139 (49.5)	0.002
Papillary thyroid carcinoma	118 (77.6)	124 (89.2)	0.011
Conventional variant	61 (51.7)	51 (41.1)	0.121
Follicular variant	18 (15.2)	28 (22.6)	0.189
Papillary microcarcinoma	23 (19.5)	26 (20.9)	0.873
Encapsulated variant	0	1 (0.8)	-
Oncocytic variant	0	2 (1.6)	-
Warthin-like variant	3 (2.5)	6 (4.8)	0.500
Tall cell variant	7 (5.9)	6 (4.8)	0.780
Hobnail variant	3 (2.5)	2 (1.6)	0.677
Solid-trabecular variant	3 (2.5)	0	-
Diffuse sclerosing variant	0	2 (1.6)	-
Follicular thyroid carcinoma	1 (0.80)	0	-
Hürthle cell carcinoma	0	2 (1.4)	-
Poorly differentiated thyroid carcinoma	8 (5.2)	5 (3.6)	0.577
Anaplastic thyroid carcinoma	2 (1.3)	0	-
Medullary thyroid carcinoma	15 (9.9)	4 (2.9)	0.017
Metastasis	3 (1.9)	1 (0.7)	0.623
Other type **	5 (3.3)	3 (2.2)	0.725

* The parametric Fisher test was applied; the *p*-value was obtained by comparing the prevalence of different thyroid lesions/tumors in our institution between 2017 and 2019 versus 2020–2022; statistically significant differences are shown in bold ** including cases of lymphoma, angiosarcoma, plasmacytoma; NIFTP: noninvasive follicular thyroid neoplasm with papillary-like nuclear features; TT-UMP: thyroid tumor of uncertain malignant potential.

**Table 2:** Demographic characteristics of the patients included in the study, in comparison before COVID-19 (2017–2019) versus the COVID-19 (2020–2022) period, for the three study groups. The distribution of different types of benign, non-tumoral thyroid lesions is highlighted in Table 1, in comparison before COVID-19 (2017–2019) versus the COVID-19 (2020–2022) period. The most frequent lesion was nodular goiter (79% versus 84.5%), followed by autoimmune thyroid disease: autoimmune thyroiditis (7.9% versus 7.5%) and Basedow–Graves disease (7.1% versus 5.5%). Cases of diffuse goiter (3.9% versus 1%), thyroid cysts (1.5% versus 1%), or subacute thyroiditis (0.3% versus 0%) were rare. No significant differences were observed in the annual rate for any of these thyroid lesions between the two studied periods.

Pathology Group	2017–2019				2020–2022				*p*		
	Average Age (Years)	W/M Ratio	Average Age for Men (Years)	Average Age for Women (Years)	Average Age (Years)	W/M Ratio	Average Age for Men (Years)	Average Age for Women (Years)	Average Age	Average Age for Men (Years)	Average Age for Women (Years)
Benign non-tumoral thyroid pathology	53.59	9.2/1	**54.43**	53.48	53.67	7.3/1	**60.83**	52.69	0.552	**0.002**	0.556
Benign thyroid tumors	49.73	7.7/1	50.90	49.58	51.90	6.8/1	51.68	53.42	0.363	0.694	0.419
Malignant thyroid tumors	50.96	4.6/1	**54.64**	50.10	49.57	5.3/1	**48.47**	48.71	0.356	0.186	0.542

Table 2 Legend: Statistically significant differences are shown in bold

## Data Availability

The data presented in this study are available on request from the corresponding author. The data are not publicly available due to ethical restrictions (personal data protection of the patients included in the study).
